# Online Removal of Baseline Shift with a Polynomial Function for Hemodynamic Monitoring Using Near-Infrared Spectroscopy

**DOI:** 10.3390/s18010312

**Published:** 2018-01-21

**Authors:** Ke Zhao, Yaoyao Ji, Yan Li, Ting Li

**Affiliations:** 1 State Key lab Elect Thin Film & Integrated Device University of Electronic Science and Technology of China, Chengdu 610054, China; zhaoke@uestc.edu.cn (K.Z.); 2014030301002@std.uestc.edu.cn (Y.J.); 2 Biomedical Engineering Institute, Chinese Academy of Medical Science and Peking Union Medical College, Tianjin 300192, China; 3 Design Center Avic Beijing Keeven Aviation Instrument Co., Ltd., China Aviation Industry Corporation, Beijing 100098, China; liyansweet@126.com

**Keywords:** baseline shifts, fitting function, near-infrared spectroscopy, polynomial function

## Abstract

Near-infrared spectroscopy (NIRS) has become widely accepted as a valuable tool for noninvasively monitoring hemodynamics for clinical and diagnostic purposes. Baseline shift has attracted great attention in the field, but there has been little quantitative study on baseline removal. Here, we aimed to study the baseline characteristics of an in-house-built portable medical NIRS device over a long time (>3.5 h). We found that the measured baselines all formed perfect polynomial functions on phantom tests mimicking human bodies, which were identified by recent NIRS studies. More importantly, our study shows that the fourth-order polynomial function acted to distinguish performance with stable and low-computation-burden fitting calibration (R-square >0.99 for all probes) among second- to sixth-order polynomials, evaluated by the parameters R-square, sum of squares due to error, and residual. This study provides a straightforward, efficient, and quantitatively evaluated solution for online baseline removal for hemodynamic monitoring using NIRS devices.

## 1. Introduction

Recently, continuous-wave near-infrared spectroscopy (NIRS) has been widely applied in industrial [1], agricultural [2], and clinical measurements [3,4,5]. Among the applications developed so far, NIRS for detection of tissue hemodynamics has made great progress, including the device’s portability, noninvasiveness, high speed, sensitivity, and ability to analyze multiple components simultaneously. These features make NIRS a promising technique in clinics and health care [6,7,8,9,10].

NIRS collects spectrum responses that include not only information from the sample itself, but also noise from other irrelevant sources, such as noise due to variations of electrical characteristics of the sample, the sample’s particle size, light-scattering effects, or shot noise from the detector [11,12]. Thus, pretreatment to eliminate irrelevant noise is required [13]. Common pretreatment methods include mean centering, auto scaling, normalization, smoothing, standard normal variate transformation (SNV), detrending, multiplicative scatter correction (MSC), and orthogonal signal correction (OSC) [11,13,14,15]. SNV was adopted to correct variations in density and particle size [16]. MSC and extended MSC are used to calibrate baseline shift arising from scattering effects in the spectrum measurements due to a change in wavelength [13]. The detrending method is always used to eliminate baseline shift in a diffuse reflection spectrum. For clinical applications, NIRS measurements are very sensitive to baseline shift, since the collected hemodynamic signal is weak. Since the measurements can be extensively affected by even a small baseline shift, the detrending method plays a vital role in removing baseline shift for clinical NIRS measurements.

Previous NIRS studies on using the detrending method to remove baseline shift primarily concentrated on the chemistry, food, and industrial fields [11]. It was demonstrated to be a feasible tool to improve the accuracy of component quantification, such as lactate concentration, poultry muscle defects, and prediction of petroleum product models [17,18,19,20]. However, few studies have looked at calibrating baseline shift for in vivo hemodynamic chromosphere measurements [21]. The Savitzky Golay (SG) algorithm has been used to smooth the data and eliminate baseline shift effects in industrial chemistry and brain-computer interface [13,15,22]. Other studies have reported the feasibility of the second-derivative approach to quantify baseline changes by fitting dynamic data to peak-wavelength response in the absorption spectrum [23]. Some recent NIRS studies on brain function initially reported using detrending methods, which were mainly focused on polynomial function fitting in data preprocessing [24,25]. However, none of the reasons or quantitative evaluations supporting the polynomial fitting calibration method were provided [24,25]. Taken together, baseline shift has attracted broad attention, especially functional NIRS in neuroscience [26], but no study so far has proposed an effective, easy-to-use, and quantitatively evaluated online calibration of baseline shift for in vivo hemodynamics monitoring with NIRS instruments. 

Unlike spectrum measurements with varying wavelengths calibrated by SNV, MSC, or the extended MSC method to remove scattering variations, we measured optical density changes over time at three specific wavelengths that are not fit to be calibrated by the above methods. The reported baseline removal method used in hemodynamics-monitoring NIRS is actually mainly polynomial fitting. Thus, our study focused on polynomial fitting for baseline removal and we attempted to find the optimal order polynomial fitting for our baseline function. 

Here, we collect and mathematically characterize baseline shift in a standard phantom test using mimicked human bodies. With such a characterized function, we were able to subtract baseline shift online and calibrate the hemodynamic measurements by NIRS accordingly. First, we used a noninvasive clinic-specific NIRS device developed in our lab to collect data with a solid phantom as the reference standard [27,28,29,30,31]. Then, the reference baseline was extracted and fitted with a series of mathematical functions provided in the MATLAB curve fitting toolbox. The highest level/value of fitting function was picked up and analyzed. Finally, we evaluated functions characterizing baselines with varied human blood hemodynamics experiments.

## 2. Materials and Methods

### 2.1. Detrending Method

The detrending method is usually used to eliminate the baseline shift of the diffuse reflectance of an SNV-processed spectrum. It has also been used to eliminate the effect of the offset generated by sensors or later quantification. This method can be used alone without SNV. It is a direct way to subtract an optimal fitting linearity, plane, or surface from an original spectrum so that the trends caused by sensors and samples can be calibrated [11,16].

The initial step of detrending is to trace the trend. Typically, the first- or second-order polynomial is adjusted to fit the trend [32]. Some studies have proposed that higher-order polynomials may help obtain a better level/value of fit, but with more risk of removing factual information [11]. For densely packed samples, the trend becomes curvilinear instead of linear. In this case, a second-order polynomial is mostly used as the following [16]:

(1) $$f\left(x\right)=p_{1}x^{2}+p_{2}x+p_{3}$$

where $p_{1}$, $p_{2}$, and $p_{3}$ are the constant coefficients of the second-order polynomial.

### 2.2. Solid Phantom

Homogeneous phantoms are widely used for NIRS calibration [33]. We chose a solid phantom that is principally composed of polyvinyl alcohol gel. The gel provides absorptivity and scattering coefficients that are close to those of the human body. Enclosed by a latex shell, the gel is mixed with titanium dioxide microsphere particles (14 ± 7 µm diameter; refractive index *n* ≈ 1.58 at 780 nm) that can scatter [27]. The solid phantom is shaped into a 6-sided rectangle with the dimensions 100 mm × 85 mm × 44 mm. This phantom, perfect for mimicking the human body, is reproducible and stable for years, and is usually used to evaluate NIRS performance [27].

### 2.3. Data Acquisition System

**Figure 1:**
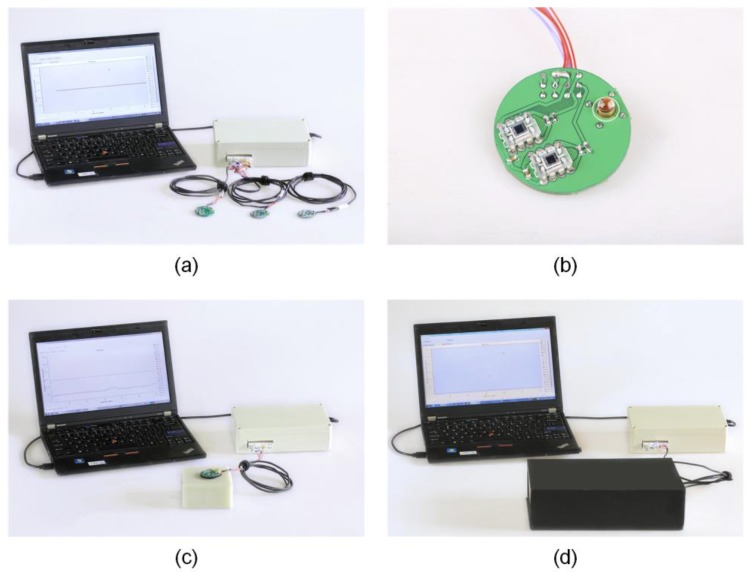
Near-infrared spectroscopy (NIRS) device. (**a**) Three parts of the device: the software in a computer, the three probes, and the functional module; (**b**) a probe with one light-emitting diode (LED) source and two sensors; (**c**) a device with the probe stuck on the solid phantom; (**d**) a device working with the probe and phantom inside a full dark box.

The system consists of 3 parts: the software in a computer, 3 probes, and the functional module (Figure 1a) [28]. The software was coded in C/C++ and LabWindows/CVI, with the functionalities of collecting data, translating to hemodynamic information, and displaying the data in real time. Each probe is composed of 1 LED source and 2 photo sensors, forming 2 channels in total (Figure 1b). At wavelengths of 735 nm, 805 nm, and 850 nm, we used the EPITEX L735.805.850-40C32P LED as the light source, which is a widely accepted choice for hemodynamics detection with NIRS [29,30]. OPT101 was chosen as the sensor, and the integrating preamplifier has good features of high optical signal conversion linearity, low output noise, and small dark current [30].

The functional module is the key part of the device. It includes a power circuit, control module, light source drive, data conversion module, display module, and transmission module [31]. The brief working process starts from the control module. Then the photo diodes receive attenuated emerging light. The data conversion module translates the light intensity data into hemodynamic data. Ultimately, the communication module sends the data to the software, where the waveforms are generated, displayed, and stored. 

### 2.4. Baseline Extraction and Removal

A soft probe-shaped black sticker is attached to the probe to ensure that the probe is tightly placed together with the sample and the background light is kept away. Probe 1 was placed on the surface of the solid phantom (Figure 1c). Then we placed Probe 1 and the phantom in a dark box. The software in the computer started to collect light intensity data for >3.5 h (Figure 1d). We performed this experiment with Probe 2 and Probe 3 in turn [28]. All the collected data from the 3 probes were then imported into MATLAB to perform function fitting. We used the R-square value and the sum of squares due to error of prediction (SSE) value to evaluate how well it fit by using the fitting function, and to find the optimal function [34]. SSE is the sum of squares due to error given by Equation (2), which represents the extent the fitting function gets to.

(2) $$\mathrm{SSE}=\overset{n}{\underset{i=1}{\sum }}w_{i}{\left(y_{i}-{\hat{y}}_{i}\right)}^{2}$$

$y_{i}$ is the response value, while ${\hat{y}}_{i}$ is the corresponding predicted value. $w_{i}$ is the weight to minimize the error estimate as a scale factor. A value of SSE closer to 0 indicates a better fit.

The R-square value represents the determination coefficient given by Equation (5), which refers to the level of fitting effect. R-square is defined as the ratio of the sum of squares of the regression (SSR) and the total sum of squares (SST). SSR is defined as

(3) $$\mathrm{SSR}=\overset{n}{\underset{i=1}{\sum }}w_{i}{\left({\hat{y}}_{i}-\bar{y}\right)}^{2}$$

SST is the sum of squares about the mean, given as

(4) $$\mathrm{SST}=\overset{n}{\underset{i=1}{\sum }}w_{i}{\left(y_{i}-\bar{y}\right)}^{2}$$

where SST=SSR+SSE. Given these definitions,

(5) $$R-\mathrm{square}=\frac{SSR}{SST}=1-\frac{SSE}{SST}.$$

A value of R-square closer to 1 indicates a better fit.

### 2.5. Experiment on Removal Effect

To evaluate calibration effectiveness by the optimal fitting function, a blood model experiment was performed and baseline removal was carried out online. This study was approved by the ethics committee (approval no. XHECD-2014-005). Sixty healthy volunteers (32 men and 28 women) from the university community were recruited by the affiliated hospital of our university, Sichuan Provincial People’s Hospital. The average age was 22.9 years for both genders. No volunteer had taken any drugs before researchers drew 5 mL blood samples. All volunteers provided written consent to participate in this study. One woman and 2 men were excluded because of blood-related diseases that could affect the results. The details of the experimental setup and protocol have been fully described in reference 32. Briefly, we structured a cylindrical phantom container into which human blood was injected. Then we injected yeast to deoxidize and alternately bubble oxygen gas into the container and recorded the hemodynamic variations with our NIRS device. The hemodynamic variation data, based on quantifications with different extinction coefficients, were collected to work with function fitting [35].

## 3. Results

### 3.1. Fourth-Order Polynomial Function

Data collected from three probes were extracted for fitting with MATLAB. We tried all functions offered in the curve fitting toolbox, and found the polynomial function had the best match with the highest level of fit by the following equation:

(6) $$f\left(x\right)=p_{1}x^{n}+p_{2}x^{n-1}+p_{3}x^{n-2}+\cdots +p_{n-1}x^{2}+p_{n}x+p_{n+1}$$

where $p_{1}$, $p_{2}$, $p_{3}$, ⋯, $p_{n-1}$, $p_{n}$, and $p_{n+1}$ are the constant coefficients of the n order polynomial.

This finding agreed with prior studies [13,16]. We then used polynomial functions from the second order to the fifth order. We found that the fourth- and fifth-order polynomial functions had good fitting effect. Both were close and had no significant overfitting. Since the fifth-order polynomial fitting required more computation burden than the fourth-order polynomial fitting, and the fourth-order polynomial fitting had almost the same perfect fit, we decided to use fourth-order polynomial fitting for online calibration of NIRS measurements, which had not been employed in previous studies.

**Figure 2:**
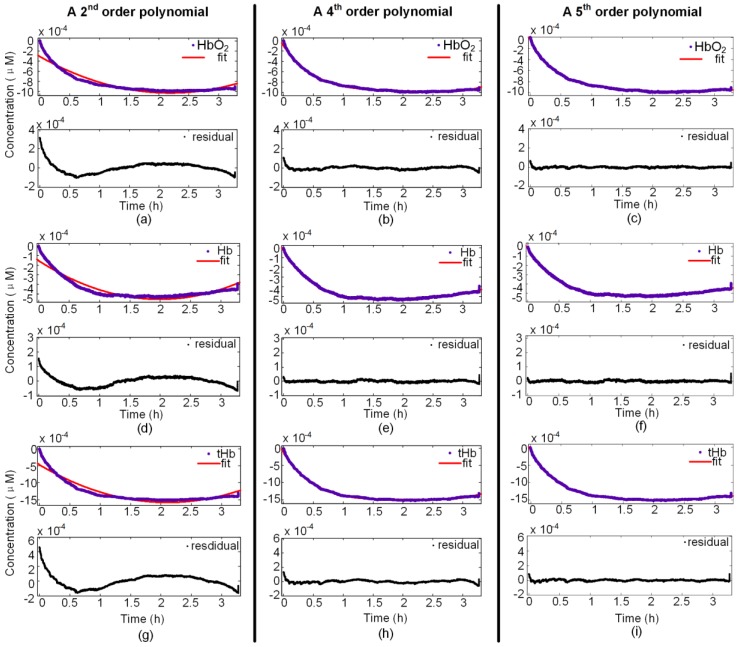
Comparisons of goodness of fit among second-order, fourth-order, and fifth-order polynomials for Probe 3 Channel 1. HbO_2_: oxygenated hemoglobin; Hb: deoxygenated hemoglobin; tHb: total hemoglobin; fit: fit data.

A comparison of the level of fitting among the fourth-order polynomial, the fifth-order polynomial, and the widely used second-order polynomial is shown in Figure 2. The typical probe, Probe 3 Channel 1, was taken as an example. The left axes (Figure 2a,d,g) are spectra that used the second-order polynomial, whereas the middle axes (Figure 2b,e,h) used the fourth-order polynomial and the right axes (Figure 2c,f,i) are spectra that used the fifth-order. Each pair of graphs consists of the original hemodynamic parameters and fit at the top, and the residuals at the bottom. Blue dots represent original data and red dots show the fit in the top graph. Black dots represent the residuals between the original data and fit. Apparently, the fit in the middle and on the right is closer to the original hemodynamic parameters than the fit on the left, and this demonstrates that the fourth-order and fifth-order polynomial functions fit better. Clearly, the residuals in the middle and right graphs (Figure 2b,c,e,f,h,i) are closer to zero and more stable than those from the second-order polynomial (Figure 2a,d,g). From the perspective of fitting a curve, the fourth-order and fifth-order polynomials undoubtedly provide better fit.

### 3.2. Evaluating the Level of Fit

Table 1 shows a quantification of the level of fit among the second-, fourth-, and fifth-order polynomial functions for all three probes, all channels, and all three hemodynamic parameters (HbO_2_, Hb, tHb). Typically, an R-square value >0.900 is a good value.

Regarding the evaluation data, all R-square values from the fourth- and fifth-order polynomials are >0.990, which is significantly higher than those from the second-order polynomial, even though they also reached a high level >0.900. The SSE values and R-square values of the fourth- and fifth-order polynomials for every hemodynamic parameter, every channel, and every probe produce much better fitting effects than the corresponding values of the second-order polynomial.

No significant overfitting is shown in the fourth- and fifth-order polynomial fit. Of note, the SEE, R-square, and residual curves of fifth-order polynomial fit are quite close to those of the fourth-order polynomial fit, suggesting that the fourth-order polynomial fit is sufficient. Taking into account that the computational burden of fifth-order polynomial fitting is higher than fourth-order polynomial fitting, we decided to use the fourth-order polynomial fitting function for online calibration of the NIRS-based medical device.

### 3.3. Verification of the Calibration

**Figure 3:**
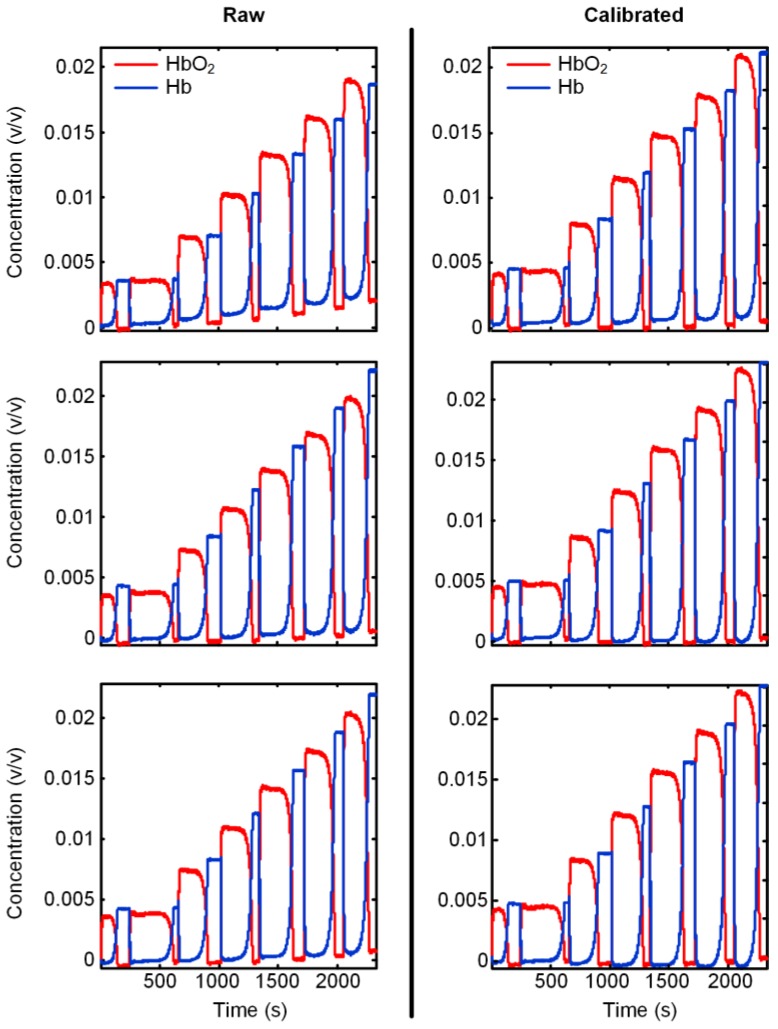
Hemodynamic parameters measured without calibration and with calibration for a typical subject as an example, using fourth-order polynomial detrending.

To verify the feasibility of fourth-order polynomial function for fitting, we performed an experiment with the function embedded system using human blood samples [35]. Data are shown with and without calibration. As shown in Figure 3, the left axes are data of HbO_2_ (oxygenated hemoglobin) and Hb (deoxygenated hemoglobin) quantified with three extinction coefficient datasets without calibration, whereas the right axes are those with calibration. The fourth-order polynomial was found to effectively remove baseline shift, successfully suppressing the primary curve downwards.

## 4. Conclusions and Discussion

The aim of this study was to explore how an optimal polynomial function would characterize baseline trend so that we could directly and effectively remove the baseline trend by subtracting the baseline-shift function online. A fourth-order polynomial fitting function is recommended, based on R-square, SSE, and residual evaluations among second- to sixty-order polynomial fittings, which showed strong potential in online baseline removal for clinical and health care NIRS instruments. This indicates that tissue HbO_2_, Hb, and tHb concentrations can be monitored in a reliable way with effective baseline removal.

For the hemodynamic parameters of NIRS, we found that the fourth-order polynomial function achieved a higher level of fit than the second-order polynomial function, which was widely used in prior studies for chemical particle or food testing with NIRS instruments [11,13,16]. All R-square values of the fourth-order polynomial function were >0.990, which was higher than those of the second-order polynomial functions (>0.900). In addition, the numerically smaller SSE values supported better fit with the fourth-order polynomial compared to the second-order polynomial. Additionally, the matches between the measured data and fitted curves and residual curves validated the perfect characterization of the fourth-order polynomial function.

Unequivocally, it is more than coincidence that all three hemodynamic concentrations attained the same improved level of fit via the experiment on removal effect. We integrated the recommended function into the software of the medical NIRS instrument for stable online calibration in a series of successful clinical applications, including shock patient monitoring [28], blood thrombosis monitoring [36], and other clinical studies [29,31]. The calibrating algorithm online for every hemodynamic parameter will improve the accuracy of medical NIRS instruments.

**Figure 4:**
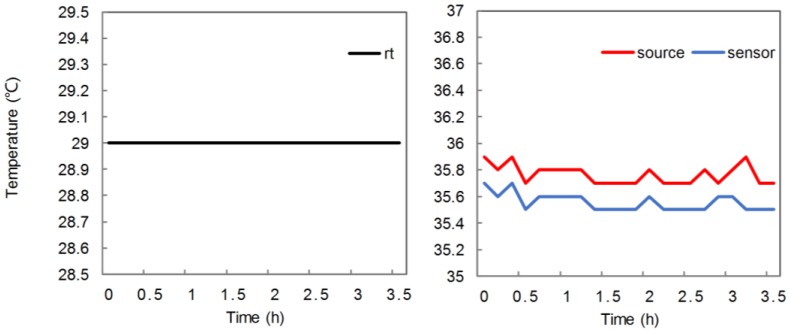
Thermal changes in typical LED source and photo sensor, Probe 3 Channel 1.

Of note, the cause of the baseline shift may not yet be clear. Originally, we guessed that baseline shift might be caused by thermal changes in LED sources and sensors. We performed a temperature test to verify this guess, using a thermometer to measure the temperature variations of both devices every 10 min for 3.5 h. Figure 4 demonstrates that the temperature change is relatively slight and does not form a shaped curve comparable to that of NIRS measurement baselines. Thus, the thermal change may not be the main cause of NIRS baseline shift. Baseline shift may depend on the algorithm of the control IC, the structure of the peripheral circuits, and the sensors. Improvement and optimization of the hardware, such as the sensors or circuits, could possibly be beneficial for reducing baseline shift.

In summary, we have characterized the baseline of hemodynamic monitoring using NIRS by fitting it with mathematical functions and determined the optimal one by full quantitative comparison in terms of the level of fitting R-square and SSE, and residual curves. We found that the fourth-order polynomial function acted the best, characterizing baselines with high reliability, effectiveness, and stability. Furthermore, calibration with this fourth-order polynomial function was successfully verified by a human blood-based varied hemodynamics experiment. The calibration approach we propose is useful for NIRS applications in clinics and health care, with merits in effectiveness, stability, speed, and convenience.

## Figures and Tables

**Table 1:** Goodness of fit statistics for hemodynamics parameters from the three probes with two different channels.

P	C	HP	Second Order		Third Order		Fourth Order		Fifth Order	
			SSE	R-Square	SSE	R-Square	SSE	R-Square	SSE	R-Square
1	1	HbO_2_	4.67 × 10^−4^	0.977	4.25 × 10^−4^	0.98	2.07 × 10^−4^	0.99	8.3 × 10^−5^	0.996
		Hb	1.65 × 10^−4^	0.979	1.27 × 10^−4^	0.981	7.81 × 10^−5^	0.99	3.05 × 10^−5^	0.996
		tHb	1.17 × 10^−3^	0.978	9.63 × 10^−4^	0.979	5.34 × 10^−4^	0.99	2.08 × 10^−4^	0.996
	2	HbO_2_	1.66 × 10^−3^	0.979	1.54 × 10^−4^	0.983	5.90 × 10^−4^	0.993	2.26 × 10^−4^	0.997
		Hb	1.87 × 10^−4^	0.975	1.59 × 10^−4^	0.981	3.26 × 10^−5^	0.996	1.71 × 10^−5^	0.998
		tHb	8.01 × 10^−4^	0.979	7.29 × 10^−4^	0.982	3.77 × 10^−4^	0.99	1.47 × 10^−4^	0.996
2	1	HbO_2_	3.87 × 10^−4^	0.957	3.38 × 10^−4^	0.958	3.52 × 10^−5^	0.996	8.08 × 10^–6^	0.999
		Hb	6.97 × 10^−4^	0.957	6.37 × 10^−4^	0.961	5.60 × 10^−5^	0.997	1.26 × 10^−5^	0.999
		tHb	4.57 × 10^−5^	0.955	3.34 × 10^−5^	0.963	4.38 × 10^−7^	0.998	5.43 × 10^−7^	0.999
	2	HbO_2_	1.60 × 10^−3^	0.977	1.29 × 10^−3^	0.979	1.61 × 10^−4^	0.998	3.78 × 10^−5^	0.999
		Hb	2.96 × 10^−4^	0.961	2.43 × 10^−4^	0.967	1.87 × 10^−5^	0.998	5.52 × 10^–6^	0.999
		tHb	5.43 × 10^−4^	0.982	4.03 × 10^−4^	0.986	8.97 × 10^−5^	0.997	3.45 × 10^−5^	0.999
3	1	HbO_2_	4.67 × 10^−4^	0.977	2.21 × 10^−4^	0.927	2.07 × 10^−4^	0.99	8.3 × 10^−5^	0.996
		Hb	1.65 × 10^−4^	0.979	6.29 × 10^−5^	0.913	7.81 × 10^−5^	0.99	3.05 × 10^−5^	0.996
		tHb	1.17 × 10^−3^	0.978	5.23 × 10^−4^	0.917	5.34 × 10^−4^	0.99	2.08 × 10^−4^	0.996
	2	HbO_2_	1.66 × 10^−3^	0.979	3.34 × 10^−4^	0.959	5.90 × 10^−4^	0.993	2.26 × 10^−4^	0.997
		Hb	1.87 × 10^−4^	0.975	1.43 × 10^−4^	0.968	3.26 × 10^−5^	0.996	1.71 × 10^−5^	0.998
		tHb	8.01 × 10^−4^	0.979	4.03 × 10^−4^	0.95	3.77 × 10^−4^	0.99	1.47 × 10^−4^	0.996

P: probe; C: channel; HP: hemodynamic parameters.
